# Histone deacetylases 1, 2 and 3 are highly expressed in prostate cancer and HDAC2 expression is associated with shorter PSA relapse time after radical prostatectomy

**DOI:** 10.1038/sj.bjc.6604199

**Published:** 2008-01-22

**Authors:** W Weichert, A Röske, V Gekeler, T Beckers, C Stephan, K Jung, F R Fritzsche, S Niesporek, C Denkert, M Dietel, G Kristiansen

**Affiliations:** 1 Institute of Pathology, Charité – Universitätsmedizin, Berlin, Germany; 2 Therapeutic Area Oncology, Nycomed GmbH, Konstanz, Germany; 3 Department of Urology, Charité – Universitätsmedizin, Berlin, Germany; 4 Institute of Surgical Pathology – University Hospital Zurich, Zurich, Switzerland

**Keywords:** HDAC, prostate cancer, prognostic marker, immunohistochemistry

## Abstract

High activity of histone deacetylases (HDACs) causes epigenetic alterations associated with malignant cell behaviour. Consequently, HDAC inhibitors have entered late-phase clinical trials as new antineoplastic drugs. However, little is known about expression and function of specific HDAC isoforms in human tumours including prostate cancer. We investigated the expression of class I HDACs in 192 prostate carcinomas by immunohistochemistry and correlated our findings to clinicopathological parameters including follow-up data. Class I HDAC isoforms were strongly expressed in the majority of the cases (HDAC1: 69.8%, HDAC2: 74%, HDAC3: 94.8%). High rates of HDAC1 and HDAC2 expression were significantly associated with tumour dedifferentiation. Strong expression of all HDACs was accompanied by enhanced tumour cell proliferation. In addition, HDAC2 was an independent prognostic marker in our prostate cancer cohort. In conclusion, we showed that the known effects of HDACs on differentiation and proliferation of cancer cells observed *in vitro* can also be confirmed *in vivo*. The class I HDAC isoforms 1, 2 and 3 are differentially expressed in prostate cancer, which might be important for upcoming studies on HDAC inhibitors in this tumour entity. Also, the highly significant prognostic value of HDAC2 clearly deserves further study.

Prostate cancer is the most common malignant neoplasm in men and ranks second only to lung cancer as a cause of tumour-related death of males in the United States (Jemal *et al*, 2007). Although a majority of patients have a relatively good prognosis after primary treatment with prostatectomy or irradiation, this disease entity also comprises a subgroup of highly aggressive neoplasms with a dismal prognosis. So far, the estimation of the individual patient's prognosis is, despite considerable investigative efforts, unreliable, which underscores the necessity of novel prognostic markers (Andren *et al*, 2006). Organ-confined tumours might be treated curatively with surgery, radiotherapy and hormonal ablation alone; however, with the onset of metastases, the disease will eventually take a lethal course (Carroll, 2005). Quite clearly, novel treatment strategies complementing the therapeutic arsenal of surgeons and oncologists in the fight against this neoplasm are urgently needed.

Recently, an entirely new group of chemotherapeutics has emerged, which target the enzyme family of histone deacetylases (HDACs) and thus change the epigenetic configuration of tumour cells (Li *et al*, 2005; Yoo and Jones, 2006). These substances, called histone deacetylase inhibitors (HDIs), possess marked antineoplastic properties and have entered clinical trials for a broad variety of malignant tumours including prostate cancer (Zhang *et al*, 2006). To date, four HDAC classes comprising 18 isoenzymes are known. Histone deacetylases are responsible for the deacetylation of histone tails, which leads to a tighter wrapping of the DNA around the histone core and consequently alters gene transcription (Minucci and Pelicci, 2006). Additionally, inhibition of deacetylation of a variety of proteins implicated in tumorigenesis by HDIs might further contribute to the antitumour effects of these substances (Mie Lee *et al*, 2003; Drummond *et al*, 2005; Floryk and Huberman, 2005; Roy *et al*, 2005). Inhibition of class I and class II members of this enzyme family by HDIs, such as valproic acid (VPA) and suberoylanilide hydroxamic acid (SAHA), causes growth arrest, differentiation and/or apoptosis of tumour cells (Richon *et al*, 2001; Blaheta *et al*, 2005) and may dramatically enhance radiation-induced apoptosis (Chinnaiyan *et al*, 2005). Although some HDIs, including SAHA and VPA, are in late-phase clinical trials, it is surprising to learn how little is known about the contribution of specific HDAC isoforms to the tumorigenic potential of HDACs.

In this study, we aimed to determine the expression patterns of HDAC1, 2 and 3 in prostate cancer, using a large clinically well-characterized patient cohort to clarify a diagnostic or prognostic value of selected class I HDACs.

## PATIENTS, MATERIALS AND METHODS

### Patient characteristics

One hundred and ninety-two patients (age: 46–73 years, median 62.5 years) who were diagnosed for prostate cancer at the Institute of Pathology, Charité – Universitätsmedizin Berlin, after radical prostatectomy, between 1991 and 2001, were included in this study. The study has been approved by the Charité University Ethics Committee under the title ‘Retrospektive Untersuchung von Gewebeproben mittels immunhistochemischer Färbung und molekularbiologischer Methoden’ (‘*Retrospective analysis of tissue samples by immunohistochemistry and molecular biological techniques*’) (EA1/06/2004) on 20 September 2004.

PSA levels were used as clinical surrogate markers for preoperative tumour volume (preoperative PSA, data available for 156 patients) and time to relapse (time course of postoperative PSA, data available for 150 patients). For those patients for whom all necessary data were available (*n*=124), Kattan scores and probability of 7-year disease-free survival (DFS) were calculated using the respective postoperative nomogram for patients after radical prostatectomy (Kattan *et al*, 1999).

None of the patients in our cohort received chemotherapy or hormonal therapy before surgery. After prostatectomy, none of the patients received adjuvant hormonal therapy; hormonal therapy was usually initiated only when a relapse occurred. Patients with a pT3 tumour and apical R1 situation received local postoperative radiotherapy. Clinical follow-up data were available for 150 patients. A PSA recurrence, which was indicative of progression of prostate cancer, was defined as a persistent increase of PSA from the nadir value of ⩽0.04 ng ml^−1^. The median follow-up time of patients still relapse-free at the end of analysis was 50 months (mean: 61.9 months).

### Tissue and clinicopathological data

All prostatectomy specimens were completely embedded and were reviewed to establish stage and grade of the respective prostate cancers. For the construction of our cohort, prostate tissue blocks that contained large areas of prostate cancer and normal prostate parenchyma were selected. Gleason sums were condensed into a ‘low-grade’ (2–6), ‘intermediate-grade’ (7) and ‘high-grade’ (8–10) group for further analysis. The distribution of clinicopathological data in the study cohort is given in Table 1.

### Immunohistochemistry

For immunohistochemical detection of HDAC isoforms on tissue samples, prediluted (‘ready to use’) polyclonal rabbit IgG antibody directed against HDAC1 (1 : 11; Abcam, Cambridge, UK), monoclonal mouse IgG antibody directed against HDAC2 (1 : 5000; Abcam) and monoclonal mouse IgG antibody directed against HDAC3 (1 : 500; Becton Dickinson, Franklin Lakes, NJ, USA) were used on 3 *μ*m paraffin sections after a standard heat-induced antigen retrieval as previously described (Noske *et al*, 2005).

Ki-67 (MIB-1) staining was performed with a Ventana autostainer (Ventana, Tucson, AZ, USA) using a monoclonal mouse IgG antibody (1 : 50; Dako, Glostrup, Denmark) under standard conditions.

### Evaluation of staining of tissue slides

Nuclear staining of HDAC isoforms was scored by applying a semiquantitative immunoreactivity scoring (IRS) system that incorporates the percentual area and the intensity of immunoreactivity, resulting in a score ranging from 0 to 12, as described (Noske *et al*, 2005). Two clinical pathologists (WW and GK) independently scored the cases. Carcinomas and prostatic intraepithelial neoplasia (PIN), if present, were scored separately. Differences in the evaluation were discussed at a multiheaded microscope until consensus was reached. For statistical analysis, cases exhibiting an IRS from 0 to 6 were lumped in an HDAC low group, whereas cases with a higher IRS (7–12) were designated HDAC high group.

The Ki-67 index was determined by counting Ki-67-positive tumour cell nuclei per 100 tumour cells in a representative, carefully selected tumour area. Mean proliferative activity of prostate carcinomas was 8.6% (s.d.: 6.62%) of tumour cells and thus quite low in comparison to other solid human malignancies.

### Statistical analysis

Statistical analyses were performed with SPSS 14.0 and GraphPad Prism 4.0. Fisher's exact and *χ*^2^-tests were applied to assess the statistical significance of the associations between expression of HDACs and clinicopathological parameters. Immunoreactivity scores for HDAC expression in PIN and carcinomas were correlated by Spearman's rank order correlation. The Mann–Whitney *U*-test was used to compare Kattan scores and normogram probability of DFS in different HDAC expression groups. Unpaired *t*-test was used to compare Ki-67 levels. Univariate survival analysis was carried out according to Kaplan–Meier, differences in survival curves were assessed with the log-rank test. The Cox regression model was used for multivariate survival analysis. *P*-values <0.05 were considered significant.

## RESULTS

### Expression patterns of class I HDAC isoforms in prostate tissue

**Figure 1:**
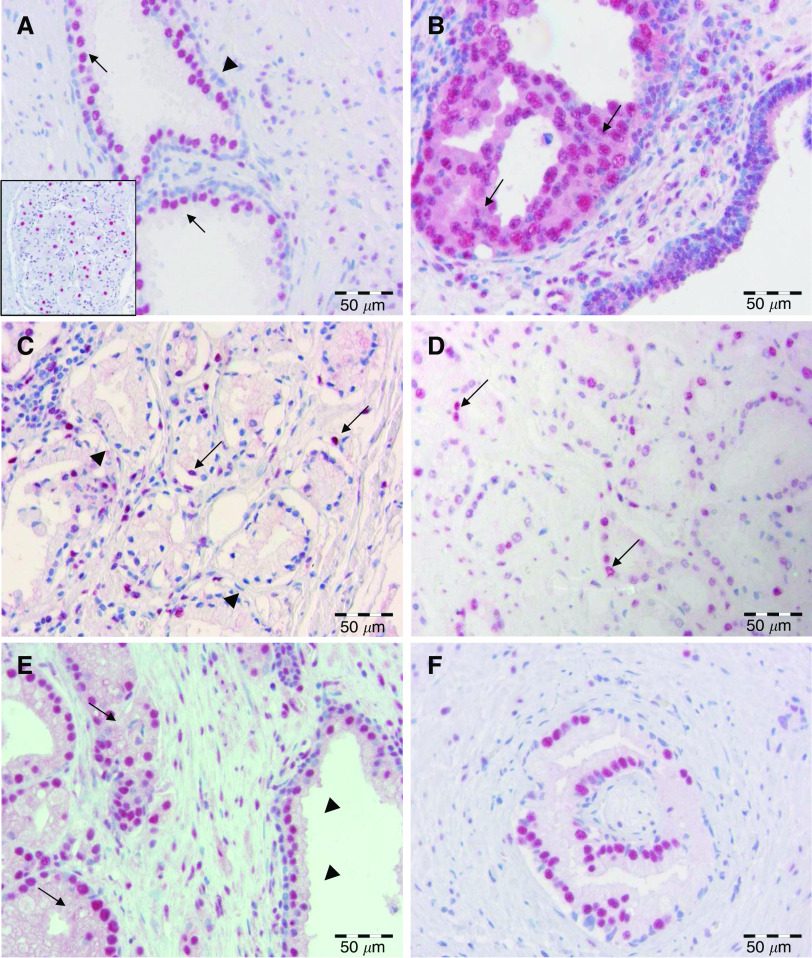
Class I HDAC expression in prostate tissue. (**A**) Moderate HDAC3 staining was evident in the nuclei of luminal epithelial cells (arrows) of normal prostate glands, whereas the majority of basal epithelial cells revealed only weak HDAC positivity (arrowhead). Note occasional moderate HDAC3 expression in prostate stroma cells. Inset: autonomous neural plexus cells exhibiting strong HDAC3 nuclear staining. (**B**) High-grade PIN (arrows) with strong nuclear positivity for HDAC2. (**C**) Microacinar prostate adenocarcinoma with only few tumour cell nuclei showing moderate expression of HDAC2. This case was scored as HDAC2 low. (**D**) Prostate carcinoma with moderate nuclear expression of HDAC1 (arrows) in approximately 70% of tumour cells. (**E**) Adenocarcinoma with homogenous strong nuclear expression of HDAC2 (arrows). Note adjacent normal prostate gland (arrowheads). (**F**) Perineural microacinar neoplastic infiltrates exhibiting strong HDAC3 expression in approximately 70% of tumour cell nuclei.

Normal prostate tissue in the vicinity of prostate carcinomas was evaluated for all 192 cases. HDAC staining of normal prostate tissue showed a characteristic pattern with only minimal variation between cases: nuclei of stromal cells of normal prostate parenchyma displayed a discontinuous weak to moderate expression of HDAC1, HDAC2 and HDAC3. Luminal epithelial cells of normal prostate glands showed a homogenous moderate positivity for all three HDAC isoforms; basal cells were mostly negative (Figure 1, Supplementary Figures S1–S3).

Strong nuclear HDAC1, HDAC2 and HDAC3 immunoreactivity was seen in most adenocarcinomas (Figure 1, Supplementary Figures S1–S3). However, staining intensity and the number of cells stained varied depending on the HDAC isoform investigated. Of 192 cases, 134 (69.8%) and 142 (74%) cases were scored high for HDAC1 and HDAC2, respectively, whereas an even higher percentage of cases (94.8%, 182 cases) showed a strong nuclear positivity for HDAC3 (Table 1). In three cases, additional cytoplasmic positivity, exclusively seen in HDAC2 immunostainings, was observed in a minority of tumour cells (Supplementary Figure S2). Completely negative cases (IRS 0) were not observed for HDAC1, HDAC2 and HDAC3, indicating that a maintained expression of these proteins is important for prostate cancer. Expression of HDAC isoforms showed a high degree of concordance (*P*<0.01, Table 1), suggesting a shared regulation. Stromal cells of prostate cancers also displayed weak to moderate nuclear positivity for all the three HDAC isoforms, which was mostly due to positive staining in fibroblasts. If present, scattered admixed inflammatory cells were positive, as well. Slight variations in stromal HDAC expression between different carcinoma cases on the one hand and carcinoma and normal parenchyma on the other hand were noted but not scored.

Lesions of high-grade PIN in the vicinity of invasive carcinomas were identified in 56 (HDAC1), 57 (HDAC2) and 67 (HDAC3) cases, respectively (Figure 1, Supplementary Figures S1–S3). High expression of HDACs was noted in 43 (76.8%), 36 (63.2%) and 66 (98.5%) cases for HDAC1, HDAC2 and HDAC3, respectively. Expression of HDACs in high-grade PIN was almost identical with the strength of expression in the corresponding invasive carcinomas (HDAC1: *r*=0.961, *P*<0.001, HDAC2: *r*=0.756, *P*<0.001, HDAC3: *r*=0.694, *P*<0.001), indicating that HDAC overexpression is an early event in prostate carcinogenesis.

### Correlation of HDAC isoform expression with clinicopathological factors and survival

HDAC1 (*P*=0.006) and HDAC2 (*P*=0.047) expression but not HDAC3 (*P*=0.584) expression correlated positively with Gleason scores (Table 1), with high-grade tumours expressing both isoforms at higher rates. In addition, expression of HDAC1 (*P*=0.032), HDAC2 (*P*=0.002) and HDAC3 (*P*<0.001) correlated significantly with the Ki-67-positive proliferative fraction of prostate cancer cells (Table 1). This indicates that the interlink between HDAC activity and cell differentiation, as well as cell proliferation, which has been proposed on the basis of cell culture models of human tumours, can be measured and thus confirmed in human prostate cancer.

To determine the probability of 7-year DFS, the postoperative Kattan nomogram was applied. Probability of postoperative DFS was lower in the HDAC1 high *vs* HDAC1 low group (median Kattan score: 183 *vs* 163, median DFS probability: 0.6 *vs* 0.8) and in the HDAC2 high *vs* HDAC2 low group (median Kattan score: 183 *vs* 154, median DFS probability: 0.6 *vs* 0.83) but not in the HDAC3 high *vs* HDAC3 low group (median Kattan score: 175 *vs* 181, median DFS probability: 0.7 *vs* 0.6) (Table 1). However, only the differences for HDAC2 were statistically significant (score: HDAC1: *P*=0.260, HDAC2: *P*=0.034, HDAC3: *P*=0.979; DFS probability: HDAC1: *P*=0.203, HDAC2: *P*=0.036, HDAC3: *P*=0.946) (Table 1).

**Figure 2:**
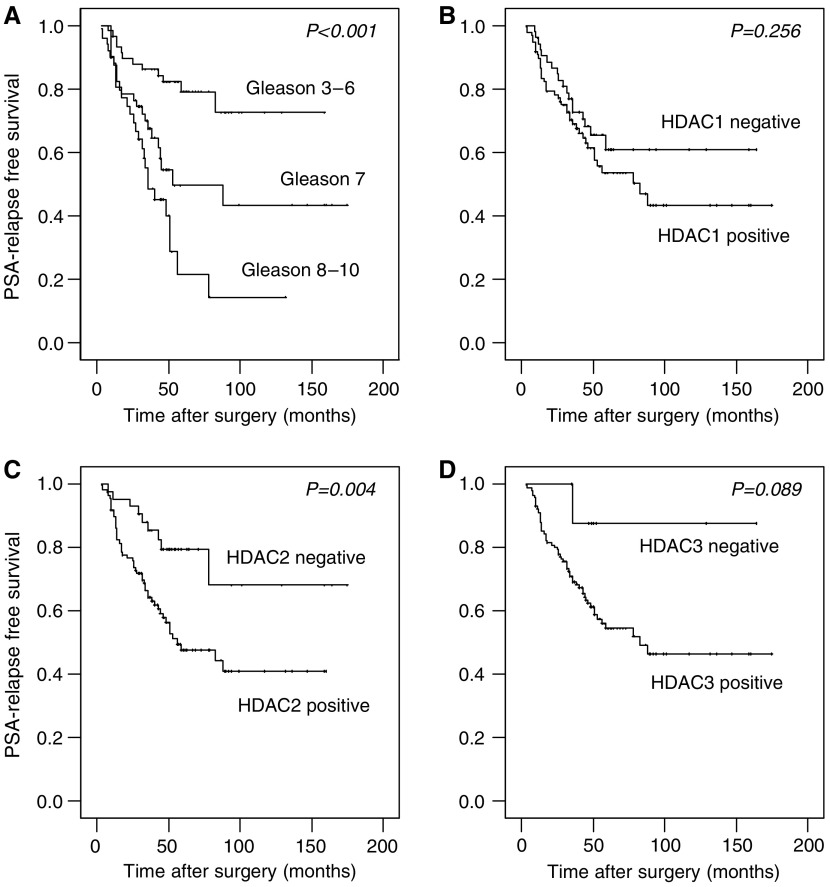
Kaplan–Meier survival curves in dependence of Gleason grade and class I HDAC expression patterns. PSA-relapse-free survival in dependence of (**A**) Gleason grade, (**B**) HDAC1, (**C**) HDAC2 and (**D**) HDAC3 expression. *P*-values were calculated with the log-rank test.

Univariate survival analysis in our cohort demonstrated that patients with high HDAC1, 2 and 3 expression were prone to earlier disease relapse (Table 2, Figure 2). However, statistical significance was reached only for HDAC2. By stratifying patients for Gleason groups, we found that differences in relapse-free survival in dependence of HDAC2 expression were especially prominent in the subgroup of patients with Gleason 7 tumours (Gleason 2–6: *P*=0.833, Gleason 7: *P*=0.008, Gleason 8–10: *P*=0.272).

The effect of HDAC2 on patient prognosis became even more pronounced in the multivariate survival analysis (Table 3) under inclusion of stage, grade and status of resection margins and preoperative PSA, which demonstrated an independent prognostic significance of HDAC2 expression (*P*=0.02, Hazard ratio=2.4). In a multivariate survival analysis under additional inclusion of the proliferative fraction in the subgroup of patients for whom data on Ki-67 index were available, HDAC2 expression retained its prognostic significance (*P*=0.03, Supplementary Table S1).

## DISCUSSION

This is the first comprehensive immunohistochemical analysis of the expression of several class I HDAC proteins (1, 2 and 3) in prostate cancer. In our study, we found all the three isoforms highly expressed in the majority of cancer cases and in the corresponding PIN lesions, indicating that upregulation of class I HDACs is an early event in prostate carcinogenesis. We assume that increased expression of these proteins is accompanied by increased overall activity. This view is supported by the fact that clear-cut correlations between expression patterns and several clinicopathological factors, as well as patient survival, were evident for selected isoforms. However, whether mutations might impair the activity of overexpressed HDACs in prostate cancer must be clarified in further studies.

High expression levels of class I HDACs correlated with tumour dedifferentiation and higher proliferative fractions (measures by Ki-67) in prostate carcinoma, which is in line with *in vitro* studies, which showed that high HDAC activity leads to tumour dedifferentiation and enhanced tumour cell proliferation (Munster *et al*, 2001; Floryk and Huberman, 2005; Uchida *et al*, 2005).

Expression of HDAC isoforms have been analysed by Waltregny *et al* (2004) in prostate cancer cells and a small set of prostate cancer tissue on mRNA and protein level. In their study, the authors did not find differences of HDAC1 expression between normal and malignant prostate tissue. In contrast, Halkidou *et al* (2004) reported an overexpression of HDAC1 protein in neoplastic prostate tissue, which was especially pronounced in hormone refractory prostate cancers. This is basically in line with our finding of higher HDAC levels in more aggressive tumours, even though the tumours of our cohort represent untreated primaries, most of which are supposedly hormone-naïve. Apart from a study on HDAC1 and HDAC3 expression in breast cancer describing an overexpression of both isoforms (Krusche *et al*, 2005), reports on expression patterns of distinct HDAC isoforms in tumour entities are sparse and most of the cohorts investigated were of a small sample size.

Our survival analyses clearly demonstrate that high HDAC2 expression is associated with shortened patient relapse-free survival time in prostate cancer, which is especially prominent in the clinically important and prognostically heterogeneous subgroup of patients with Gleason 7 carcinomas. This not only hints at an important role of this isoform in prostate cancer progression but also suggests HDAC2 expression as a novel prognostic marker for prostate cancer. Our findings are supported by a significant lower probability of DFS for patients with HDAC2 high tumours when compared with patients with HDAC2 low tumours as calculated by the respective Kattan nomogram. However, the finding that HDAC2 is an independent prognosticator in prostate cancer ought to be verified in a larger prospective study. Technically, we found the evaluation of HDAC2 immunostainings rather straightforward, as the percental nuclear immunoreactivity was relatively independent of varying antibody concentrations. This is a prerequisite for a robust semiquantitative evaluation and is likely to reduce interlab variability and makes HDAC2 an interesting candidate prognostic marker for further validation.

It is possible that the prognostic value of HDAC2 expression is based on its strong correlation with the proliferative fraction. However, uncontrolled cell proliferation is only a single but not the decisive feature of malignancy. Other effects of aberrant HDAC expression in prostate cancer relevant to tumorigenesis, such as altered cell migration (Rombouts *et al*, 2002; Klisovic *et al*, 2005), invasive potential or increased angiogenesis (Kim *et al*, 2001, 2004; Kwon *et al*, 2002; Qian *et al*, 2006) might contribute to the dismal prognosis of patients with HDAC2 high tumours, but this is so far not proven experimentally for this tumour entity. Also, in a multivariate Cox analysis with inclusion of the Ki-67 fraction, HDAC remained an independent prognostic factor, whereas Ki-67 failed significance.

The fact that HDAC1 expression correlated strongly with tumour dedifferentiation indicates a prominent role of this isoform in the control of prostate tumour differentiation. The observation that HDAC1 nonetheless had no significant impact on patient prognosis might be explained by the fact that malignant tumour behaviour is a composition of the above-mentioned plethora of factors and is not determined by differentiation alone.

The high percentage of HDAC3 positivity (95%) in prostate cancer naturally compromised valid correlations and survival analyses but may make this isoform interesting in terms of a therapy target.

Functionally, a growth inhibition of prostate cancer cells by administration of HDIs has already been shown both *in vitro* and in animal models (Butler *et al*, 2000; Kuefer *et al*, 2004; Thelen *et al*, 2004; Fronsdal and Saatcioglu, 2005; Gediya *et al*, 2005; Myzak *et al*, 2006). Divergent effects of therapeutic concentrations of the HDAC inhibitors SAHA and VPA on tumour cell cycle, with the former inducing a G_2_/M arrest and the latter inducing a G_1_ arrest, were reported for other tumour cell lines as well (Takai *et al*, 2004a, 2004b). An important role of class I HDACs, especially HDAC3, on cell proliferation has also been reported for other tumour entities (Wilson *et al*, 2006), which again makes it an interesting therapy target.

Very recently, a variety of fusion genes have been discovered in prostate cancer, which appeared to be centrally involved in carcinogenesis. In this context, it should be noted that HDAC1 was associated with an upregulation of the androgen-responsive gene ERG, which results from a gene fusion of TMPRSS2 with oncogenic ETS factors (Iljin *et al*, 2006). So far, it is unknown if other class I HDAC isoforms are upregulated by genomic alterations as well.

In summary, this study demonstrated that the three class I HDAC isoforms 1, 2 and 3 are highly expressed in a considerable fraction of adenocarcinomas of the prostate. High expression levels of HDAC2 have a highly significant negative prognostic impact in terms of PSA relapse-free survival times. The consistently high rate of HDAC3 positivity in prostate cancer might be of interest for further exploratory therapeutic studies. We hypothesize that the outcome of patients who are going to be treated with HDIs being currently in clinical trials is likely to be influenced by the expression patterns of HDAC isoforms, which should be the focus of further analyses.

## Figures and Tables

**Table 1:** Overall expression of class I HDAC isoforms in prostate carcinoma as well as distribution of class I HDAC isoform expression in the study population stratified for selected tumour parameters

	**Total**	**HDAC1 low**	**HDAC1 high**	***P*-value**	**HDAC2 low**	**HDAC2 high**	***P*-value**	**HDAC3 low**	**HDAC3 high**	***P*-value**
All cases	192 (100%)	58 (30.2%)	134 (69.8%)		50 (26%)	142 (74%)		10 (5.2%)	182 (94.8%)	
PSA ⩽10 ng ml^−1^	75 (48.1%)	23 (30.7%)	52 (69.3%)	0.610^+^	23 (30.7%)	52 (69.3%)	0.474^+^	4 (5.3%)	71 (94.7%)	1.000^+^
PSA >10 ng ml^−1^	81 (51.9%)	29 (35.8%)	52 (64.2%)		20 (24.7%)	61 (75.3%)		5 (6.2%)	76 (93.8%)	
Age ⩽65	134 (69.8%)	43 (32.1%)	91 (67.9%)	0.494^+^	29 (21.6%)	105 (78.4%)	0.048^+^	8 (6%)	126 (94%)	0.726^+^
Age >65	58 (30.2%)	15 (25.9%)	43 (74.1%)		21 (36.2%)	37 (63.8%)		2 (3.4%)	56 (96.6%)	
pT2	97 (50.5%)	32 (33%)	65 (67%)	0.580^*^	28 (28.9%)	69 (71.1%)	0.398^*^	5 (5.2%)	92 (94.8%)	0.925^*^
pT3	91 (47.4%)	24 (26.4%)	67 (73.6%)		21 (23.1%)	70 (76.9%)		5 (5.5%)	86 (94.5%)	
pT4	4 (2.1%)	2 (50%)	2 (50%)		1 (25%)	3 (75%)		0 (0%)	4 (100%)	
Gleason sum 2–6	70 (36.5%)	31 (44.3%)	39 (55.7%)	0.006^*^	24 (34.3%)	46 (65.7%)	0.047^*^	5 (7.1%)	65 (92.9%)	0.584^*^
Gleason sum 7	64 (33.3%)	14 (21.9%)	50 (78.1%)		15 (23.4%)	49 (76.6%)		2 (3.1%)	62 (96.9%)	
Gleason sum 8–10	58 (30.2%)	13 (22.4%)	45 (77.6%)		11 (19%)	47 (81%)		3 (5.2%)	55 (94.8%)	
R0	100 (52.1%)	29 (29%)	71 (71%)	0.754^+^	30 (30%)	70 (70%)	0.249^+^	5 (5%)	95 (95%)	1.000^+^
R1	92 (47.9%)	29 (31.5%)	63 (68.5%)		20 (21.7%)	72 (78.3%)		5 (5.4%)	87 (94.6%)	
HDAC1 low	58 (30.2%)	—	—	—	23 (39.7%)	35 (60.3%)	0.007^+^	10 (17.2%)	48 (82.8%)	<0.001^+^
HDAC1 high	134 (69.8%)	—	—		27 (20.1%)	107 (79.9%)		0 (0%)	134 (100%)	
HDAC2 low	50 (26%)	23 (46%)	27 (54%)	0.007^+^	—	—	—	7 (14%)	43 (86%)	0.004^+^
HDAC2 high	142 (74%)	35 (24.6%)	107 (75.4%)		—	—		3 (2.1%)	139 (97.9%)	
HDAC3 low	10 (5.2%)	10 (100%)	0 (0%)	<0.001^+^	7 (70%)	3 (30%)	0.004^+^	—	—	—
HDAC3 high	182 (94.8%)	48 (26.4%)	134 (73.6%)		43 (23.6%)	139 (76.4%)		—	—	
Ki-67 index mean	8.60	6.88	9.30	0.032^#^	6.44	9.30	0.002^#^	4.00	8.83	<0.001^#^
Ki-67 index s.d.	6.62	0.82	0.63		0.61	0.64		0.63	0.53	
DFS probability median	0.70	0.80	0.60	0.203^$^	0.83	0.60	0.036^$^	0.60	0.70	0.946^$^
DFS probability quart.	0.3–0.9	0.30–0.91	0.30–0.86		0.40–0.93	0.30–0.85		0.20–0.90	0.30–0.90	

DFS=disease-free survival; HDAC=histone deacetylase.

In the first row overall distribution of the respective tumour parameters in the study population is listed.

^+^Fisher's exact test, ^*^χ^2^-test for trends, ^#^unpaired *t*-test ^$^, Mann–Whitney *U*-test.

**Table 2:** Patient survival in dependence of several clinicopathological factors and HDAC isoform expression (*n*=150)

**Characteristic**	**No. of cases**	**No. of events**	**Mean PSA-relapse-free time (±s.e.) in months**	***P*-value**
HDAC1 expression				0.256
Low	53	18	112 (±10)	
High	97	41	97 (±9)	
				
HDAC2 expression				0.004
Low	42	9	134 (±13)	
High	108	50	86 (±7)	
				
HDAC3 expression				0.089
Low	9	1	148 (±15)	
High	141	58	101 (±7)	
				
Pre-OP PSA				0.006
⩽10 ng ml^−1^	72	20	117 (±8)	
>10 ng ml^−1^	73	37	75 (±10)	
				
Age				0.336
⩽65	105	44	99 (±9)	
>65	45	15	108 (±12)	
				
pT stage				0.003
pT2	78	22	111 (±9)	
pT3/pT4	72	37	84 (±10)	
				
Histological grade (Gleason)				<0.001
Gleason sum 2–6	59	12	127 (±9)	
Gleason sum 7	51	22	95 (±12)	
Gleason sum 8–10	40	25	50 (±8)	
				
R-status				0.006
R0	79	23	122 (±9)	
R1	71	36	76 (±10)	
				
Ki-67 index				0.010
⩽10%	95	34	104 (±8)	
>10%	34	21	62 (±14)	

HDAC=histone deacetylase.

*P*-values were calculated with the log-rank test.

**Table 3:** Cox regression analysis with inclusion of HDAC2 expression (*n*=145)

	**Overall survival**		
	**HR**	**95%CI**	***P*-value**
HDAC2 expression			
Low	1.000		
High	2.363	1.150–4.856	0.019
			
Pre-OP PSA			
⩽10 ng ml^−1^	1.000		
>10 ng ml^−1^	1.542	0.884–2.691	0.127
			
Tumour stage			
pT2	1.000		
pT3/pT4	1.557	0.830–2.919	0.168
			
Gleason sum			
2–6	1.000		
7	2.795	1.325–5.898	0.007
8–10	3.131	1.403–6.989	0.005
			
R-status			
R0	1.000		
R1	1.440	0.789–2.631	0.235

CI=confidence interval; HDAC=histone deacetylase; HR=hazard ratio.
