# Supplementary figures and images for: Histone deacetylases 1, 2 and 3 are highly expressed in prostate cancer and HDAC2 expression is associated with shorter PSA relapse time after radical prostatectomy
Source: Br J Cancer. 2008 Jan 22;98(3):604–10. doi: 10.1038/sj.bjc.6604199 (PMC2243142; doi:10.1038/sj.bjc.6604199)

## Slide 1
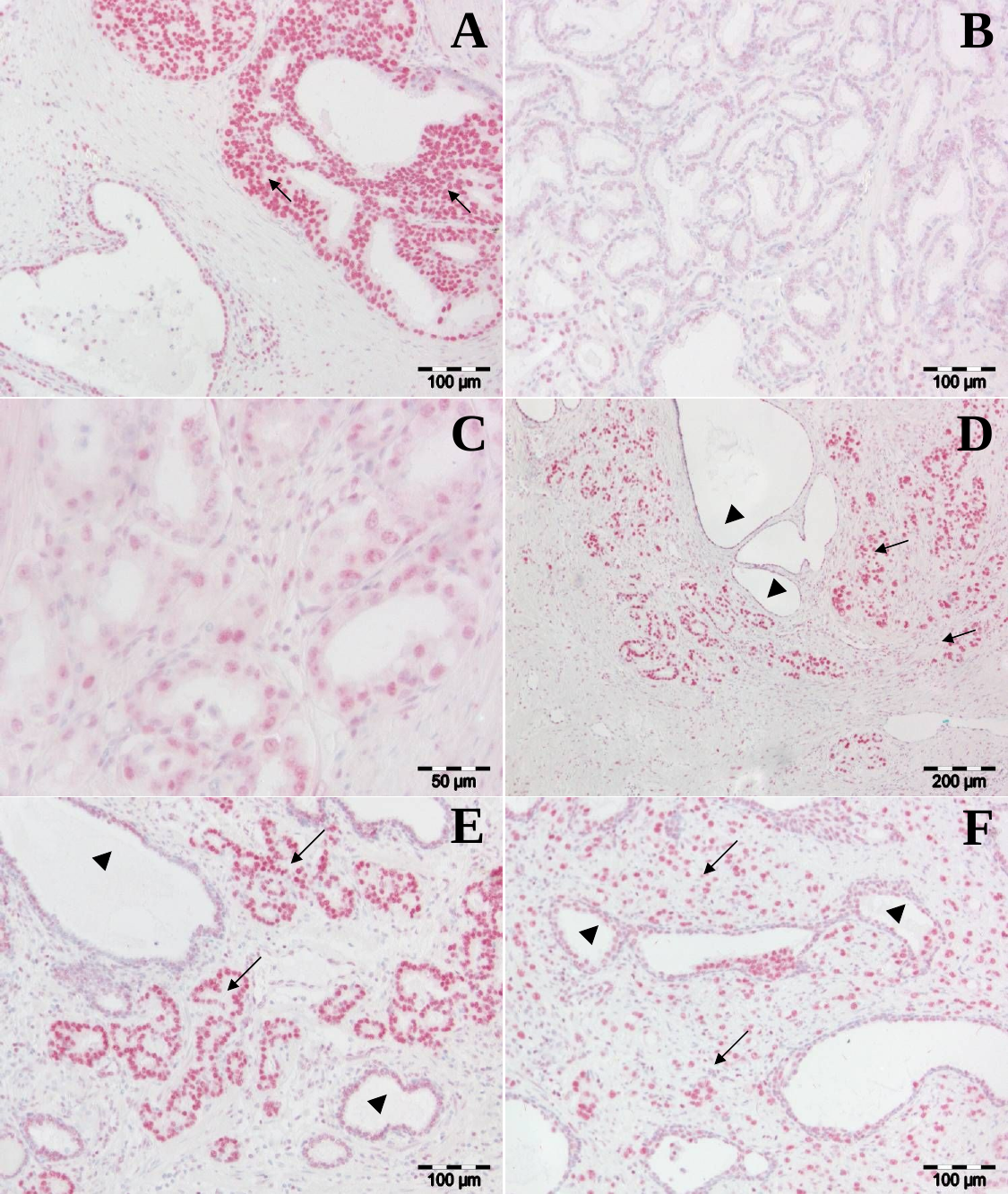

A
B
C
D
E
F
102 (66.7%)
51 (33.3%)

Supplement: Supplementary Figure S1 [file 6604199x1.ppt]

## Slide 1
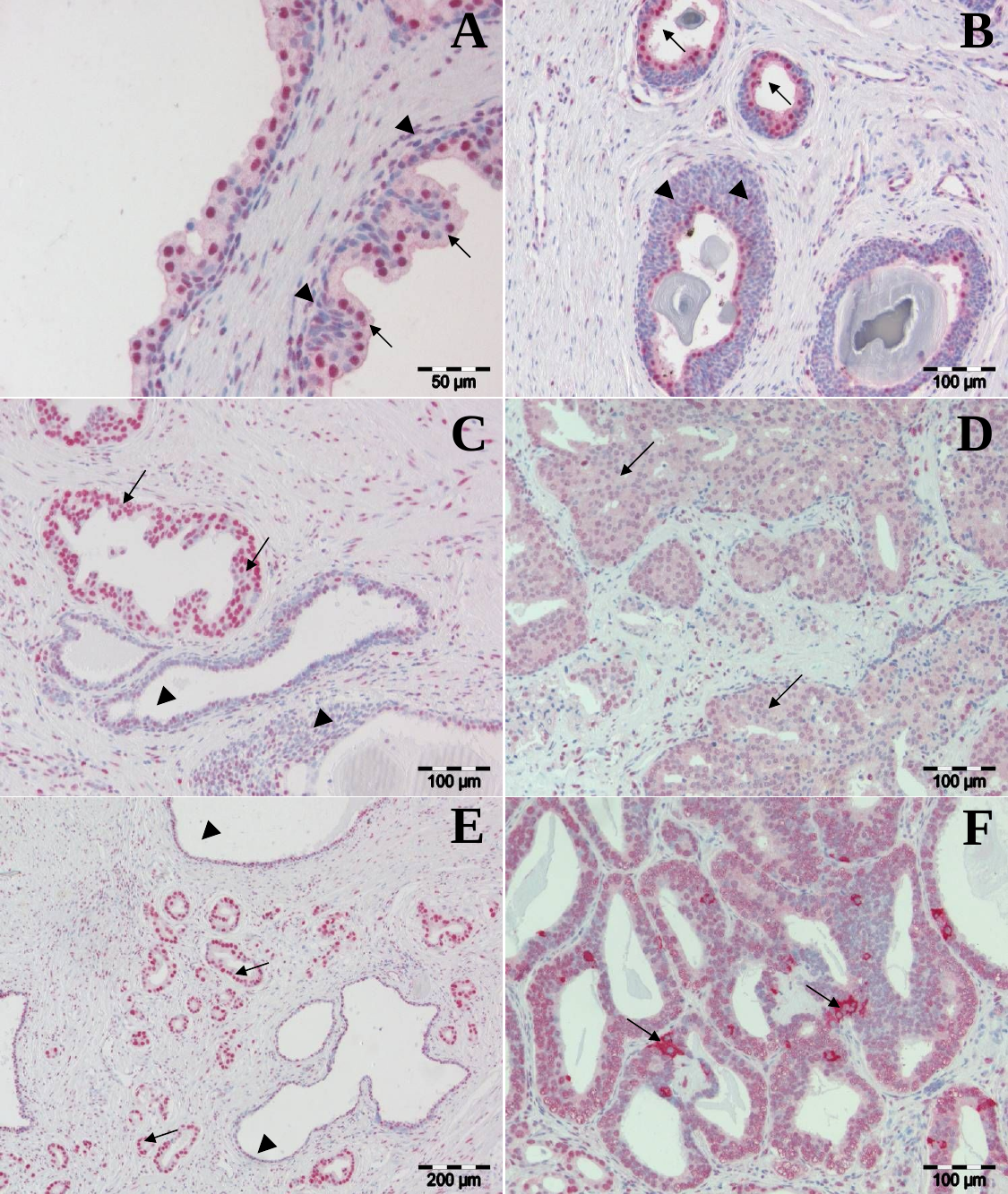

A
B
C
D
E
F
51 (33.3%)

Supplement: Supplementary Figure S2 [file 6604199x2.ppt]

## Slide 1
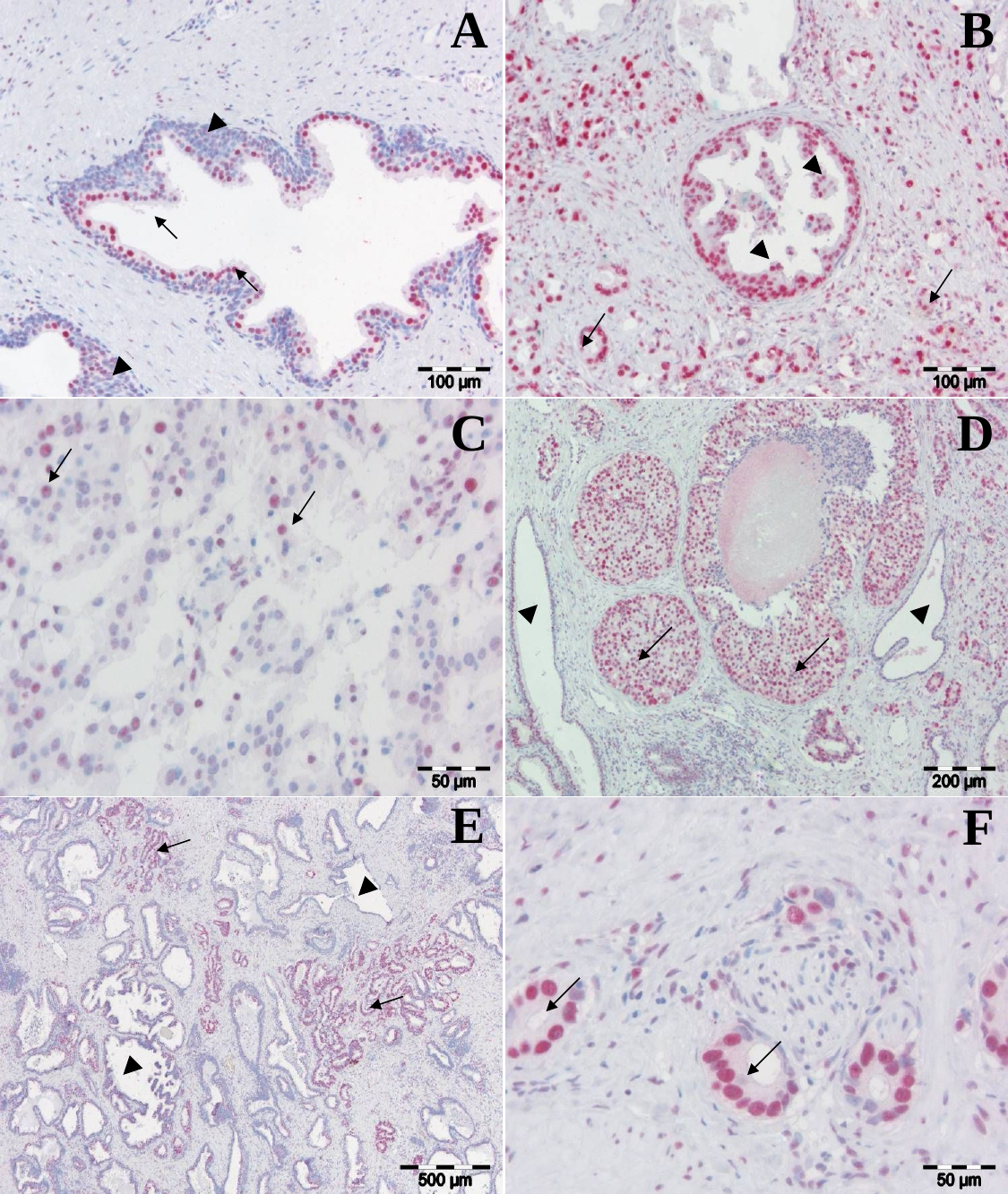

A
B
C
D
E
F
51 (33.3%)

Supplement: Supplementary Figure S3 [file 6604199x3.ppt]
