# Supplementary material for: Histone deacetylases 1, 2 and 3 are highly expressed in prostate cancer and HDAC2 expression is associated with shorter PSA relapse time after radical prostatectomy
Source: Br J Cancer. 2008 Jan 22;98(3):604–10. doi: 10.1038/sj.bjc.6604199 (PMC2243142; doi:10.1038/sj.bjc.6604199)
Supplement: Supplementary Table S1 and Figure Legends [file 6604199x4.doc]

**Table S1 Cox regression analysis with inclusion of HDAC2 expression and proliferative activity (n=124).**

|  | **Overall survival** | | |
| --- | --- | --- | --- |
|  | **HR** | **95%CI** | **p-value** |
| ***HDAC2 expression*** |  |  |  |
| negative | 1.000 |  |  |
| positive | 2.263 | 1.086-4.714 | 0.029 |
| ***Ki-67 index*** |  |  |  |
| ≤10% | 1.000 |  |  |
| >10% | 1.587 | 0.804-3.133 | 0.183 |
| ***Pre-OP PSA*** |  |  |  |
| ≤10 ng/ml | 1.000 |  |  |
| >10 ng/ml | 1.638 | 0.923-2.907 | 0.092 |
| ***Tumor stage*** |  |  |  |
| pT2 | 1.000 |  |  |
| pT3/pT4 | 1.432 | 0.740-2.770 | 0.286 |
| ***Gleason sum*** |  |  |  |
| 2-6 | 1.000 |  |  |
| 7 | 2.620 | 1.197-5.731 | 0.016 |
| 8-10 | 2.308 | 0.965-5.517 | 0.060 |
| ***R-status*** |  |  |  |
| R0 | 1.000 |  |  |
| R1 | 1.114 | 0.570-2.180 | 0.752 |

**´**

**Supplemental figure legends**

**Figure S1. HDAC1 expression in prostate tissue**

**(A)** Strong HDAC1 staining in the nuclei of a high grade PIN lesion (arrows). Low **(B)** and high **(C)** power magnification of microacinar prostate adenocarcinomas with weak nuclear expression of HDAC1. **(D/E/F)** Prostate carcinomas with strong nuclear expression of HDAC1 (arrows). Note glands with normal prostate parenchyma in the direct vicinity (arrowheads).

**Figure S2. HDAC2 expression in prostate tissue**

**(A)** Moderate nuclear HDAC2 staining in luminal epithelial cells (arrows) of normal prostate glands. Note that basal epithelial cells only weakly expressed the protein (arrowheads). **(B)** Normal prostate glands with basal cell hyperplasia. HDAC2 expression was almost absent in the hyperplastic basal cells (arrowheads). Note remaining HDAC2 positive luminal epithelial cells (arrows). **(C)** High grade PIN lesion (arrows) with strong nuclear positivity for HDAC2, note normal glands in the vicinity (arrowheads). **(D)** Prostate carcinoma (arrows) with only weak nuclear expression of HDAC2. **(E)** Adenocarcinoma (arrows) with strong nuclear expression of HDAC2. Note adjacent normal prostate glands (arrowheads). **(F)** Adenocarcinoma with weak nuclear expression of HDAC2. However, in addition to nuclear staining cytoplasmic positivity could be observed in single tumor cells as well (arrows).

**Figure S3. HDAC3 expression in prostate tissue**

**(A)** Normal prostate gland with focal basal cell hyperplasia. Note moderate HDAC3 staining in the nuclei of luminal epithelial cells (arrows) while the majority of basal epithelial cells revealed only weak HDAC3 positivity (arrowheads). **(B)** Invasive prostate carcinoma (arrows) with accompanying high grade PIN (arrowheads). Both lesions are strongly positive for HDAC3. **(C)** Prostate carcinoma with occasional moderate nuclear expression (arrows) of HDAC3 in approximately 20% of tumour cells. **(D/E/F)** Prostate adenocarcinomas (arrows) with strong nuclear expression of HDAC3. Note glands with normal prostate parenchyma in the direct vicinity (arrowheads in **D** and **E**).
